# A Global Perspective for Sustainable Highway Tunnel Lighting Regulations: Greater Road Safety with a Lower Environmental Impact

**DOI:** 10.3390/ijerph15122658

**Published:** 2018-11-27

**Authors:** Antonio Peña-García, Thi Phuoc Lai Nguyen

**Affiliations:** 1 Department of Civil Engineering and Research Group “Lighting Technology for Safety and Sustainability”, University of Granada, 18071 Granada, Spain; 2 Department of Development and Sustainability, School of Environment, Resources and Development, Asian Institute of Technology, 12120 Pathumthani, Thailand; phuoclai@ait.asia

**Keywords:** public perception, tunnel safety, environmental impacts, driver well-being, sustainability

## Abstract

Tunnel lighting installations function 24 h a day, 365 days a year. These infrastructures have increased exponentially and now connect quite distant locations, even on different continents. This has led European administrations and international regulatory bodies to establish regulations for tunnel safety with the lowest environmental impact. However, until now, these regulations have almost exclusively focused on traffic safety, and relegated sustainability to the background. Even though they recognize the need to reduce energy consumption, they do not propose any tools for doing so. Given the impact of these installations and the lack of a specific regulatory framework, Asian countries will soon be forced either to update previous standards for tunnel lighting or elaborate new ones. A better understanding of the weaknesses of European regulations combined with a willingness to embrace innovation could position Asia as a world leader in the regulation of more sustainable road tunnels. The objective of this research was to improve the sustainability of tunnel lighting installations through new regulations or amendments to existing ones, without impairing the mental well-being of users, who could potentially be affected by energy-saving measures. Accordingly, this paper presents and analyzes a broad proposal for formulating tunnel lighting regulations. The originality of this proposal lies in the fact that it integrates road safety, lower environmental impact, and user well-being. Furthermore, it is expected to broaden the perspective of regulatory bodies and public administrations with regard to tunnel installations, which would ultimately enhance their sustainability.

## 1. Introduction

Of all the areas of human activity that have an impact on sustainability and environmental policy, lighting has come to play a major role in recent years. However, given that lighting is an extremely broad area, its environmental impact has been mainly addressed from the perspective of light pollution or urban lighting. Although these sub-areas of lighting are undeniably important, there is a third area whose impact goes beyond mere consumption. When understood in its widest sense, tunnel lighting impinges on almost all the key dimensions of sustainability.

This paper begins with a brief summary of tunnel lighting and its impact, together with a state-of-the-art overview of the effect of tunnel lighting on the environment and human well-being. In the following sections of the paper, tunnel lighting and environmental perceptions are integrated in a proposal that links traffic safety, infrastructure sustainability, and user well-being. This proposal is especially relevant for Asian countries, which aspire to become world leaders in the implementation of sustainable traffic infrastructures. In this sense, Asia is an excellent model, precisely because it still has no well-established policies regarding tunnel lighting and sustainable tunnels. This means that the incipient interest and concern of Asian countries for tunnel lighting can be channeled into a high-level policy in which users also have a voice in the design of regulations.

The main objective of this research was to improve the sustainability of tunnel lighting installations through new regulations or amendments to existing ones, without impairing the mental well-being of users, who could potentially be affected by energy-saving measures. Asian countries were used as a model because of their incipient interest in tunnel regulations, which made them excellent candidates for applying our recommendations in future tunnel lighting regulations.

## 2. Socio-Environmental Impacts of Tunnel Lighting: The X-Factor

Road traffic safety has become an important public health challenge in all societies because of the rising number of fatal and disabling accidents. This increase in road traffic accidents is caused by a wide range of factors, such as unawareness of traffic rules, ineffective road engineering, and unsustainable traffic infrastructure. Although low-income and middle-income countries account for approximately 90% of traffic deaths, fatality rates in certain high-income countries have declined over the last four decades. This difference is not only due to more advanced infrastructure and technology, but also to effective legislation. Comprehensive road traffic regulations and their strict enforcement maybe a critical factor in reducing injuries and deaths, since most accidents are the result of speeding, drunk driving, and not using occupant protection measures. For example, various nations in Southeast Asia, South America, and Africa, do not have child restraint laws, seat-belt laws for all car occupants, or drunk driving laws. In fact, in many of these countries, it is legal for drivers to have a blood alcohol concentration of more than 50 g/dL. Still another issue is the lack of enforcement of traffic regulations. For example, even though Thailand has a motorcycle helmet law, most motorcyclists do not use helmets.

Although accidents in road tunnels are not more frequent than in opencast roads, driving in underground environments is more dangerous because accidents are generally more severe, given the risk of fire, collisions against walls, and the lack of space to avoid obstacles [1,2,3]. Hence, ineffective or unsustainable tunnel lighting infrastructure could cause a higher number of injuries and deaths. A variety of factors make driving in tunnels more difficult. Physiological factors include the slow adaptation of the human visual system. Drivers are obliged to go from a bright environment to a darker one, and need several minutes to recover full visual performance [4]. Other factors, which are both physiological and psychological, are also directly related to lighting and the visual performance of drivers. One example is the flicker effect [1,4], which produces a loss of concentration, headache, dizziness and other unpleasant feelings brought on by the continuous alternation of bright and dark bands on the road and walls of the tunnel.

Finally, there are psychological factors [1], whose consequences, though less tangible, are even more dangerous because they have a negative effect on driving performance when going through tunnels. These range from panic and anxiety, which can arise when approaching a tunnel whose gate is poorly illuminated (black-hole effect), to the apparent narrowing of the walls (tunnel effect).

All of these factors that impact road safety are caused by deficient lighting. Initially, it might seem that the problem could be solved with a better design of the lighting installations. Unfortunately, the solution is not that simple. Because of tunnel geometry and the variability of driver visual performance, there is no tunnel lighting that is 100% optimal for everyone. On the other hand, the more efficient the lighting installation is for visual performance, the higher its consumption of energy, raw materials, financial resources, maintenance, and CO_2_ emission production.

As an illustration, a normal tunnel can incorporate more than 200 luminaires or projectors. Each luminaire can consume up to 400–600 W during the day, when visual adaptation is most demanding. With this configuration, the annual energy consumption (without considering installation and maintenance costs) for one tunnel costs approximately 1 million US dollars. In a mountainous country like Spain, where there are more than 500 tunnels, the financial impact is very high. The situation becomes even more serious when we consider the number of very long tunnels and roads under rivers, seas, cities, and mountains chains, whose length can easily exceed 10 km. These very long underground roads (VLUR), whose number is rapidly increasing, are possible thanks to technological advances and the need for high-capacity transportation infrastructures [1].

As a result, public administrations in countries throughout the world are becoming aware of the intimate relationship between safe tunnels and economical and environmental sustainability. Given this scenario, researchers are searching for ways to reduce the lighting requirements of tunnels [5,6,7,8], and improve the efficiency of electrical installations [9,10,11,12], as well as the optical properties of the tunnel elements such as the pavement [13,14,15,16]. However, the most important milestone has been the irruption of strategies to introduce sunlight inside the tunnels themselves. These strategies are based on the total or partial shift of the tunnel zones with the highest energy consumption [17,18,19,20,21,22,23,24,25,26] and the introduction of sunlight through light-pipes or similar devices [27,28,29]. Along with the use of natural light for more sustainable tunnels, there are also new tools that evaluate the accuracy of each strategy for each tunnel [30,31].

Although these strategies have saved a great deal of money, there are still two obstacles to an effective policy for sustainable tunnels. Firstly, strategies have developed so quickly that Europe has not had sufficient time to assimilate and include them in new standards. Secondly, new tunnel-lighting standards are exclusively focused on technical parameters, with little or no regard for the well-being and safe behavior of drivers. In fact, even though the target is an optimal compromise between safety and sustainability, user perceptions and feelings are not considered. In our opinion, this could lead to impaired driving or a poor valuation of the actions of authorities.

The next section analyzes user perceptions of the environment and the actions performed to minimize waste as well as to optimize energy consumption and sustainability.

## 3. Public Perception and Pro-Environmental Behavior of Energy Efficient Tunnel Lighting

Human behavior has environmental consequences [32]. Tunnel lighting is not an exception, since it uses an excessive amount of energy and raw materials, which contribute to air pollution, solid waste accumulation, and climate change. In addition, improper tunnel lighting design affects public health since it can cause vehicle crashes in tunnels because driving performance is influenced by tunnel design factors [33]. An increasing awareness of the environmental impact of tunnel lighting has led to appeals for more sustainable technologies.

The adoption of more environmentally-friendly behavior can reduce the environmental impact by mitigating climate change and improving human health [34]. Although research has studied how tunnel lighting design can affect driver behavior in tunnels, it has rarely focused on public perceptions and pro-environmental behavior of the environmental impacts of tunnel lighting. As a result, tunnel strategies have only been based on the considerations of policy makers, engineers, and designers. Nevertheless, their assumptions regarding public perceptions, pro-environmental behavior, and the environmental impacts of tunnel lighting may be ineffective because they fail to adequately account for the key impacts on environment and human safety that the public views as important.

Responding to the global perspective of energy savings, many Asian countries have adopted intelligent solutions for energy-efficient tunnel lighting. These strategies have focused on (i) the optimal management of lighting stages; (ii) increasing the number of lighting stages: (iii) adjusting lighting levels based on traffic speed; (iv) integrating control systems; and (v) using LED technology [35]. However, public perceptions of the environmental impacts of these strategies has never been examined. In addition, the characterization of public pro-environmental behavior in tunnel lighting has never been taken into account in the design and implementation of sustainability strategies.

In this regard, public perception of tunnel lighting impacts maybe very different in Asian countries. Perceptions of new tunnel lighting installations are based on what humans learn from their environment. People then interpret their sensory impressions on the basis of their culture, knowledge, and experiences [36]. Levels of adoption of energy-efficient tunnel lighting in Asia vary from one country to another because of different national and local socio-economic political contexts. These factors often affect energy and infrastructure technology as well as policy choices. For example, China and Japan differ socially, culturally, and politically. In China, decision-making is relatively top-down [37], whereas in Japan, decision-making is more inclusive and open [38]. This evidently affects public awareness, perception, and behavior pertaining to tunnel lighting strategies. Therefore, tunnel lighting with a sustainable design requires the integration of public perception of the ways in which tunnel lighting impacts on user well-being and the environment. It is also a question of fostering public awareness and encouraging pro-environmental initiatives.

## 4. Tunnel Regulations and Sustainability: Surveying the Public about Tunnel Lighting Strategies

As part of this research, various regulations, standards, and recommendations for tunnel lighting were analyzed in order to ascertain whether they included references to environmental concerns (apart from the obvious focus on traffic safety). Their sensitivity to user perception was also examined. For example, the CIE 88:2004 recommendation [2], which is the basis for most national regulations throughout the world, devotes only one short subparagraph to possible measures for reducing the consumption of electricity by lighting installations (i.e., Section 6.4, on the use of natural light screens). The Spanish Regulation of due compliance for all projects in Spain [39] has only one second-level paragraph on energy efficiency (7.3.1.1 Energy efficiency), and a mere 18 lines on natural light screens. The only section titled “Lighting and environment” is scarcely four pages long, and does not explore the topic in any real depth. In northern European countries, daylight screens are fleetingly mentioned at the end of the standard “Road Tunnel Lighting: Common Nordic guidelines” [40].

Despite these shortcomings, European countries mainly adapt and follow the recommendations in the CIE standard [4]. Though in force since 2004, this standard is based on former versions, and was never adapted to new strategies, which only became a serious research focus after 2010. In some Asian countries, tunnel lighting regulations are much more recent or do not exist at all. Nonetheless, the exponential growth of these countries makes these regulations a necessity, as observed by various authors [41]. It goes without saying that they should be adapted to the particularities of these countries. For example, Indonesia launched its own regulation [42], which primarily targets safety, but without any mention of energy savings.

It is evident that public reactions to tunnel lighting sustainability are currently ignored when designing new strategies for highway network safety and the mitigation of environmental impacts. Many Europe countries have made significant progress in tunnel safety legislation. Indeed, Directive 2004/54/EC of the European Parliament and of the Council of 29 April 2004 on minimum safety requirements for tunnels in the trans-European road network has been transposed and incorporated in the legislation of European member states. Although these countries have taken a holistic approach, based on best practices in tunnel management, the result has been a mixed picture of compliance to safety standards for road tunnels. There is also a lack of knowledge regarding public perception of the implementation and effects of Directive 2004/54/EC, as well as a lack of public consultation to improve tunnel legislation throughout Europe.

There is thus a need to create better opportunities and more effective mechanisms. This includes expanding the scope, for public involvement in the design and implementation of tunnel lighting strategies. Public consultation has always been an integral part of the theory and practice of public policy. However, such consultation is something new in the tunnel sector, since public interest in shaping highway network tunnel strategies has never been analyzed in many Western countries. With regard to tunnel design and construction, the public comprises various groups of stakeholders: (i) those who use the highway tunnel networks; (ii) those living near highway tunnel networks; (iii) those concerned about the impacts of tunnel design and construction on the environment. Tunnel policy makers, engineers, and designers should approach the public as partners and listen to their voices. Public investment in highways and tunnel networks should reflect their concerns, and promote user safety and general well-being. This includes reducing environmental impacts to a minimum.

## 5. Proposal of Global Regulation of Tunnel lighting Installations to Guarantee Safety, Sustainability and User Well-Being: Towards a New Generation of Environmental Policies

As previously mentioned, the main regulations, standards, and recommendations for tunnel lighting are almost exclusively focused on ensuring traffic safety. Of course, this must be apriority, since tunnels are infrastructures in which accidents can be fatal. Also to be considered are personal factors, such as fear, stress, and claustrophobia, which can impair driving. Given the impact of tunnels on user behavior and mental well-being, it is paradoxical that users have never been consulted when tunnel lighting regulations were being formulated.

A parallel consideration is the fact that tunnel lighting installations consume extremely large quantities of energy, raw materials, and financial resources. They also have a high impact on the landscape and ecosystems because of emissions from their construction and operation. Although some regulations mention the use of sunlight to reduce energy consumption, they make no specific proposal that can lead to any real implementation of the latest advances presented in the introduction section. Not surprisingly, tunnel lighting installations are still far from being as sustainable as they could be.

The triangle formed by safety, sustainability, and user well-being can only be satisfactorily completed if user perceptions of energy-saving measures and environmental protection actions are taken into account. Such measures can be perceived as totally positive, as not optimal but necessary, or as safety impairing, regardless of their positive contribution to sustainability.

**Figure 1:**
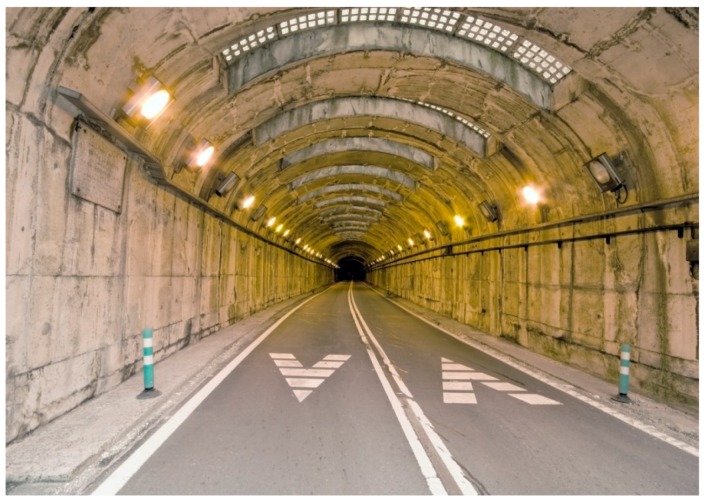
Upper windows to use natural light in an almost closed tunnel in Bielsa (Spain) (Image bought in www.istockphoto.com).

**Figure 2:**
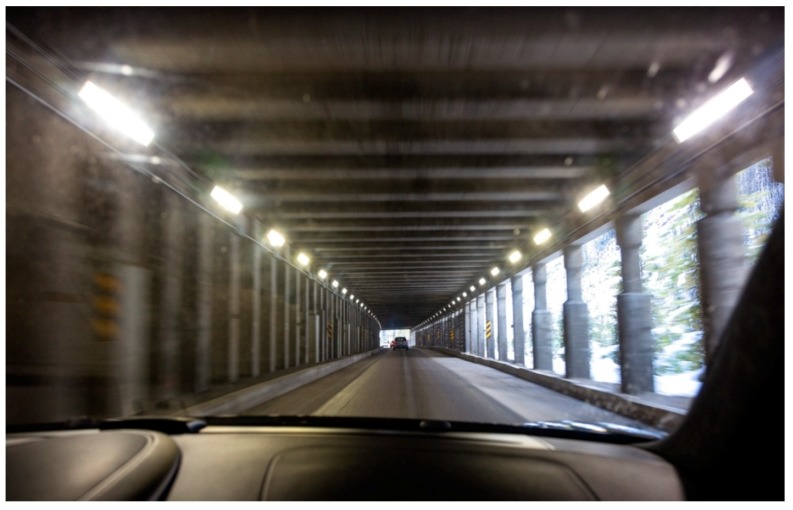
Lateral columns to use natural light in an almost open tunnel (Spain) (Image bought in www.istockphoto.com).

For example, one possible measure is the use of certain kinds of pergola to extend the most energy-consuming threshold zone of a tunnel, or upper windows (when possible) to make use of sunlight. However, these solutions generally make the tunnel longer, and thus, more dangerous, because it can cause claustrophobia (Figure 1). In contrast, other types of pergola or the suppression of one lateral wall when possible may be perceived as positive or neutral for driving safety (Figure 2).

In spite of the interest in placing pergolas before portal gates for energy savings of 30–50% [18,19], Figure 1 and Figure 2 show that different tunnel availability strategies can vary when their potential impact on user behavior and mental well-being is considered. Evidently, benefits from energy savings will be cancelled out if drivers are frightened by them, choose longer routes, or have more accidents because of perceived insecurity.

For this reason, users should be consulted if tunnels are ever to become an effective tool for sustainable development as understood in the context of the Brundtland report (“sustainable development must meet the needs of current generations without compromising the ability of future generations to meet their own needs”, [43]).

In this scenario, our proposal for formulating new regulations and updating existing ones rests on three pillars:

(i) Prioritization of driver safety through the study of the complex visual task of tunnel driving. For this reason, suitable luminance, and uniformity and limitation of glare and the flickering effect will continue to be obligatory factors as they have been up until now.
(ii) Introduction of technical annexes that list, describe, and calculate the parameters of the auxiliary installations that allow the use of sunlight (light-pipes, pergolas etc.) or which lead to lower energy needs, such as the forestation of surroundings and other similar strategies.
(iii) Research through surveys, focus groups, or other data-collection tools in order to have a realistic idea of user perception of the potential impact of these strategies. This research should seek to balance user preferences in terms of perceived safety and potential benefits in terms of energy savings and contribution to sustainability.

Even though all of these pillars are important, only the first one has been implemented, since it has always been regarded as a priority. The timeline for carrying out the rest of the proposal should be sequential. The first step would be to decide which strategies for the reduction of the environmental impact of lighting installations should be prioritized. The second stage would focus on consulting the public to obtain user perceptions of these strategies. After verifying their feasibility, road tunnels could thus become an example of fair and sustainable environmental policy, which could be used as a model by the rule-making bodies for other infrastructures. Given the relatively recent incorporation of tunnel lighting regulations in certain Asian countries, these nations would be an ideal scenario for testing this proposal.

## 6. Conclusions

This paper has discussed the current regulations, recommendations, and standards for tunnel lighting from the perspective of environmental sustainability and user perception. The results of this analysis reflected a very low concern for new strategies that use sunlight in tunnels as well as for others that reduce the consumption of energy and raw materials (with a savings of approximately 30%). In a parallel way, none of the standards analyzed considered user perceptions and opinions about the use of these strategies in tunnels, as well as their potential impact on both real and perceived safety. Given the potential influence of these strategies on tunnel users and their safety, our proposal recommends that public opinion be considered in the elaboration of new tunnel lighting regulations. Asian regulatory bodies are now in the process of developing such regulations and/or updating current ones. Their concern for these important issues and willingness to address them could make this region a world leader in the sphere of sustainable tunnels and user satisfaction though always keeping safety as the main priority.

Notwithstanding, the path towards 100% sustainable tunnels is long. Future research should be interdisciplinary and should focus on topics such as the following:

- The impact of tunnel lighting on public health and the environment.
- The integration of sociological, psychological, and engineering issues in the design and construction of sustainable tunnels.

## References

[1] Peña-García A. (2018). The impact of lighting on drivers well-being and safety in very long underground roads: New challenges for new infrastructures. Tunn. Undergr. Space Technol..

[2] Ma Z.L., Shao C.F., Zhang S.R. (2009). Characteristics of traffic accidents in Chinese freeway tunnels. Tunn. Undergr. Space Technol..

[3] Bassan S. (2016). Overview of traffic safety aspects and design in road tunnels. IATSS Res..

[4] Commision Internationale de l’Éclairage, CIE (2004). Guide for the Lighting of Road Tunnels and Underpasses.

[5] López J.C., Grindlay A.L., Carpio M.C., Peña-García A. (2014). Strategies for the optimization of binomial energy saving landscape integration in road tunnels. WIT Trans. Ecol. Environ..

[6] Peña-García A., López J., Grindlay A. (2015). Decrease of energy demands of lighting installations in road tunnels based in the forestation of portal surroundings with climbing plants. Tunn. Undergr. Space Technol..

[7] García-Trenas T., López J.C., Peña-García A. (2018). Proposal to forest Alpine tunnels surroundings to enhance energy savings from the lighting installations. Towards a standard procedure. Tunn. Undergr. Space Technol..

[8] Salata F., Golasi I., Peña-García A. (2018). Financial and environmental impact of combined actions in road tunnels for the decrease of energy and raw materials consumption. WIT Trans. Ecol. Environ..

[9] Wang X., Zhou J. (2009). The application of stepless intelligent control system to LED illuminating brightness in city tunnel. Transp. Sci. Technol..

[10] Salata F., Golasi I., Poliziani A., Futia A., de Lieto Vollaro E., Coppi M., de Lieto Vollaro A. (2016). Management Optimization of the Luminous Flux Regulation of a Lighting System in Road Tunnels. A First Approach to the Exertion of Predictive Control Systems. Sustainability.

[11] Qin L., Dong L.L., Xu W.H., Zhang L.D., Leon A.S. (2017). An Intelligent Luminance Control Method for Tunnel Lighting Based on Traffic Volume. Sustainability.

[12] Qin L., Dong L., Xu W., Zhang L., Yan Q., Chen X. (2017). A “vehicle in, light brightens; vehicle out, light darkens” energy-saving control system of highway tunnel lighting. Tunn. Undergr. Space Technol..

[13] Salata F., Golasi I., Bovenzi S., de Lieto Vollaro E., Pagliaro F., Cellucci L., Coppi M., Gugliermetti F., de Lieto Vollaro A. (2015). Energy Optimization of Road Tunnel Lighting Systems. Sustainability.

[14] Moretti L., Cantisani G., Mascio P.D. (2016). Management of road tunnels: Construction, maintenance and lighting costs. Tunn. Undergr. Space Technol..

[15] Moretti L., Cantisani G., Di Mascio P., Caro S. (2017). Technical and economic evaluation of lighting and pavement in Italian road tunnels. Tunn. Undergr. Space Technol..

[16] Moretti L., Mandrone V., D’Andrea A., Caro S. (2017). Comparative “from cradle to gate” Life Cycle Assessments of Hot Mix Asphalt (HMA) Materials. Sustainability.

[17] Gil-Martín L.M., Peña-García A., Hernández-Montes E., Espín-Estrella A. (2011). Tension structures: A way towards sustainable lighting in road tunnels. Tunn. Undergr. Space Technol..

[18] Peña-García A., Gil-Martín L.M., Escribano R., Espín-Estrella A. (2011). A Scale Model of Tension Structures in Road Tunnels to Optimize the Use of Solar Light for Energy Saving. Int. J. Photoenergy.

[19] Peña-García A., Escribano R., Gil-Martín L.M., Espín-Estrella A. (2012). Computational optimization of semi-transparent tension structures for the use of solar light in road tunnels. Tunn. Undergr. Space Technol..

[20] Peña-García A., Gil-Martín L.M. (2013). Study of pergolas for energy savings in road tunnels. Comparison with tension structures. Tunn. Undergr. Space Technol..

[21] Gil-Martín L.M., Gómez-Guzmán A., Peña-García A. (2015). Use of diffusers materials to improve the homogeneity of sunlight under pergolas installed in road tunnels portals for energy savings. Tunn. Undergr. Space Technol..

[22] Abdul Salam A.O., Mezher K.A. Energy Saving in Tunnels Lighting using Shading Structures. Proceedings of the 2014 International Renewable and Sustainable Energy Conference (IRSEC).

[23] Wang B.L., Ye Y., Yan B. (2015). Experimental Research on Application of the Natural Light to Tunnel Lighting Engineering. J. Light Vis. Environ..

[24] Drakou D., Burattini C., Bisegna F., Gugliermetti F. Study of a daylight “filter” zone in tunnels. Proceedings of the IEEE 15th International Conference on Environment and Electrical Engineering (EEEIC).

[25] Drakou D., Celucci L., Burattini C., Nardecchia F., Gugliermetti F. Study for optimizing the daylight “filter” in a pre-tunnel structure. Study for optimizing the daylight “filter” in a pre-tunnel structure. Proceedings of the IEEE 16th International Conference on Environment and Electrical Engineering (EEEIC).

[26] Drakou D., Burattini C., Mangione A., Bisegna F. Exploring the daylight simulation of filter panels in a pre-tunnel structure. Proceedings of the 2017 IEEE International Conference on Environment and Electrical Engineering and 2017 IEEE Industrial and Commercial Power Systems Europe (EEEIC/I&CPS Europe).

[27] Gil-Martín L.M., Peña-García A., Jiménez A., Hernández-Montes E. (2014). Study of Light-pipes for the use of sunlight in road tunnels: From a scale model to real tunnels. Tunn. Undergr. Space Technol..

[28] Qin X., Zhang X., Qi S., Han H. (2015). Design of Solar Optical Fiber Lighting System for Enhanced Lighting in Highway Tunnel Threshold Zone: A Case Study of Huashuyan Tunnel in China. Int. J. Photoenergy.

[29] Peña-García A., Gil-Martín L.M., Hernández-Montes E. (2016). Use of sunlight in road tunnels: An approach to the improvement of light-pipes’ efficacy through heliostats. Tunn. Undergr. Space Technol..

[30] Peña-García A. (2017). The SLT equation: A tool to predict and evaluate energy savings in road tunnels with sunlight systems. Tunn. Undergr. Space Technol..

[31] López J.C., Grindlay A.L., Peña-García A. (2017). A proposal for evaluation of energy consumption and sustainability of road tunnels: The sustainability vector. Tunn. Undergr. Space Technol..

[32] Gardner G.T., Stern P.C. (2002). Environmental Problems and Human Behavior.

[33] Kircher K., Ahlstrom C. (2012). The impact of tunnel design and lighting on the performance of attentive and visually distracted drivers. Accid. Anal. Prev..

[34] Clayton S., Devine-Wright P., Stern P.C., Whitmarsh L., Carrico A., Steg L., Swim J., Bonnes M. (2015). Psychological research and global climate change. Nat. Clim. Chang..

[35] Lim H.S., Ngarambe J., Kim J.T., Kim G. (2018). The Reality of Light Pollution: A Field Survey for the Determination of Lighting Environmental Management Zones in South Korea. Sustainability.

[36] Nguyen T.P.L., Seddaiu G., Virdis S.G.P., Tidore C., Pasqui M., Roggero P.P. (2016). Perceiving to learn or learning to perceive? Understanding farmers’ perceptions and adaptation to climate uncertainties. Agric. Syst..

[37] Lo K. (2014). A critical review of China’s rapidly developing renewable energy and energy efficiency policies. Renew. Sustain. Energy Rev..

[38] Mah D., van der Vleuten J., Hills P., Tao J. (2012). Consumer perceptions of smart grid development: Results of a Hong Kong survey and policy implications. Energy Policy.

[39] Government of Spain (2015). Ministerio de Fomento. OC 36/2015 Sobre Criterios a Aplicar en la Iluminación de Carreteras a Cielo Abierto y Túneles. Tomo II: Recomendaciones para la Iluminación de Túneles (OC 36/2015 on Criteria to be Applied in the Lighting of Opencast Roads and tunnels. Volume II: Recommendations for the Lighting of Tunnels).

[40] Nordisk VejtekniskForbund (1995). Road Tunnel Lighting: Common Nordic Guidelines.

[41] Liu H.Y. (2005). Design Criteria for Tunnel Lighting.

[42] Departemen Pekerjaan Umum (2009). Geometri Jalan Bebas Hambatan untuk Jalan Tol: Standar Kontstruksi dan Bangunan.

[43] World Commission on Environment and Development (1987). Our Common Future.

